# Challenges and recent advances in methods for handling imbalanced multiclass classification problems: a methodological review

**DOI:** 10.1186/s12874-026-02899-w

**Published:** 2026-06-20

**Authors:** Sachin Acharya, Satyanarayana Poojari, Vani Lakshmi R., Asha Kamath

**Affiliations:** 1 Department of Applied Statistics and Data Science, Prasanna School of Public Health, Manipal Academy of Higher Education, Manipal, Karnataka 576104 India; 2 Department of Health Technology and Informatics, Prasanna School of Public Health, Manipal Academy of Higher Education, Manipal, Karnataka 576104 India

**Keywords:** Binarization, Class imbalance, Multiclass classification, Multiclass imbalance, Imbalance measure, Imbalance ratio

## Abstract

**Background:**

Class imbalance is a common challenge in real-world health science applications, including medical diagnosis, rare disease detection, and ICU mortality prediction, where one or more classes are underrepresented. Although several methods address imbalance in binary classification, multiclass imbalance remains particularly challenging due to multiple minority classes, often leading to biased performance and reduced predictive accuracy. Despite several advancements, most classification models struggle to identify patterns in imbalanced data, limiting their effectiveness in real-world applications.

**Methods:**

A structured literature search was conducted to identify methodological studies on imbalanced multiclass classification, including algorithmic strategies and advances in performance evaluation. Articles published up to 2024 were retrieved from Scopus and Web of Science using predefined keywords. Studies were screened through titles and abstracts based on predefined inclusion and exclusion criteria, with additional backward citation searching for methodologically relevant studies. In total, 75 studies were included in the final methodological review to synthesize key challenges and recent advances.

**Results:**

Despite the introduction of several metrics for assessing multiclass imbalance, the Imbalance Ratio (IR) remains the most commonly used measure for quantifying imbalance severity. Existing balancing techniques mainly rely on distance-based, cluster-based, and distribution-based approaches, reflecting methodological diversity. In multiclass settings, various decomposition strategies, classification algorithms, and performance metrics have been proposed to address imbalance; however, repeated use of imbalance-handling mechanisms, such as class weight adjustments across decomposition, training, and evaluation stages, may introduce bias. The effectiveness of these strategies depends on data characteristics including dimensionality, sample size, distribution, number of classes, and imbalance severity. Notably, insufficient reporting of these characteristics in many studies limits the assessment of feasibility and generalizability across diverse data settings.

**Conclusions:**

This review synthesizes the strengths and limitations of existing methods for handling imbalanced multiclass classification, offering practical insights for improving model robustness and predictive performance. Effective management of class imbalance supports several Sustainable Development Goals by promoting equitable decision-making and enhancing reliable analysis across diverse health, societal, and environmental challenges, making it essential for developing robust and generalizable models across diverse domains.

## Background

Classification problems are commonly encountered in real-world health science applications, playing a crucial role in supporting clinical decisions and improving patient outcomes. Examples include early detection of sepsis in intensive care units, automated diagnosis of diabetic retinopathy from retinal scans, and identifying patients at high risk of cardiovascular complications. Accurate classification models in these areas can enhance patient care and support timely clinical interventions. Binary classification categorizes data into two classes, whereas multiclass classification involves more than two classes. Traditionally, multiclass problems were addressed using binarization strategies, which convert multiclass tasks into multiple binary class problems before applying classification models.

Over the years, numerous statistical and machine learning (ML) techniques have been developed to address classification tasks, typically assuming that classes are equally distributed. The primary objective of these models is to accurately predict class labels while minimizing misclassification rates.

In many real-world applications, including sentiment analysis [1], fraud detection, and medical diagnosis [2], class distributions are often imbalanced, with one or more classes underrepresented (minority) and others overrepresented (majority). Such class imbalance can result in classification models that are biased toward the majority class, leading to poor prediction of minority classes [2–3]. The problem is particularly pronounced in multiclass settings, where multiple minority and majority classes can further degrade model performance [3–4]. Class imbalance can result from intrinsic factors, reflecting the natural predominance of certain classes within a domain, or extrinsic factors, such as biased sampling or incorrect data labelling [5]. The extent of imbalance is often measured using the Imbalance Ratio (IR), which represents the ratio of majority to minority class instances. While IR is commonly applied to binary classification problems, alternative measures have been proposed to quantify imbalance in multiclass settings.

Class imbalance is commonly addressed using three strategies: data-level, algorithm-level, and hybrid approaches [2]. Hamid et al. [6] proposed a framework for handling highly imbalanced multiclass data and discussed open challenges and future directions in this area. Patel and Bhavsar [7] emphasized that multiclass imbalanced classification remains an open problem and suggested that hybrid approaches offer more effective solutions. Wang et al. [8] reported that algorithmic, ensemble, and oversampling techniques are predominantly used in the literature, with particular emphasis on handling multiclass imbalanced medical data.

Numerous methods have been proposed to manage imbalance in multiclass problems, and the effectiveness of classification models is typically evaluated using performance measures, which can assess either class-specific or overall performance. Selecting appropriate metrics is particularly critical in imbalanced scenarios, and this review highlights the performance measures commonly used for multiclass-imbalanced problems. Despite the availability of various approaches, class imbalance in multiclass classification remains a significant challenge and research in this domain has been growing rapidly due to its applicability in real-world scenarios. The novelty of this study lies in its critical review of the methods introduced in the literature to handle multiclass imbalance, highlighting their strengths and limitations, and providing a checklist of strategies for managing class imbalance in multiclass classification.

The review paper is organized as follows: Section 2 describes the detailed methodology used in the study. Section  3 includes the study results, and Section 4 provides a comprehensive discussion and conclusion based on the reviewed articles.

## Methods

This section outlines the approach used to understand class imbalance in multiclass classification problems and recent developments in this domain. Section  2.1 presents the research questions framed for this review, Section 2.2 details the search strings employed to retrieve relevant articles, and Section 2.3 describes the inclusion and exclusion criteria applied for selecting research articles.

### Research questions

With a focus on understanding the development of methodologies in the domain of multiclass imbalance problems, an attempt was made to explore the following research questions.

1. How are multiclass classification problems commonly tackled?
2. How can class imbalance be effectively measured and evaluated in multiclass classification problems?
3. How is class imbalance addressed in multiclass classification problems?
4. How is the performance of classifiers measured in the case of multiclass settings?

### Search strings

To address the above research questions, relevant articles published up to 2024 were retrieved from digital libraries Scopus and Web of Science using the following keywords.

(“multiclass” OR “multi-class” OR “multiple classes”) AND (“imbalance” OR “imbalanced” OR “unbalanced” OR “skewed”) AND (“classification” OR “classifier” OR “problem”).

### Search criteria for inclusion and exclusion of the articles

Based on the research objective, specific inclusion and exclusion criteria were applied to select relevant articles. The titles and abstracts of retrieved articles were manually screened to identify potentially relevant studies. Additionally, articles cited within the selected studies were included if they were pertinent to the research objectives, even if they did not fully meet all inclusion criteria. The detailed inclusion and exclusion criteria are outlined below.

**Table:** 

Inclusion Criteria:	Exclusion Criteria:
• Title or abstract includes keywords • Written in English language • Published in a journal or conference proceedings • Includes the development of methods	• Duplicate articles • Other languages • Unrelated topics or include topics that focus on data streams, data intrusion, concept drift, deep learning, longitudinal studies, multilabel, multi-objective, time series or mere application. • Focus on binary classification only • Retracted articles • Not accessible

**Fig. 1:**
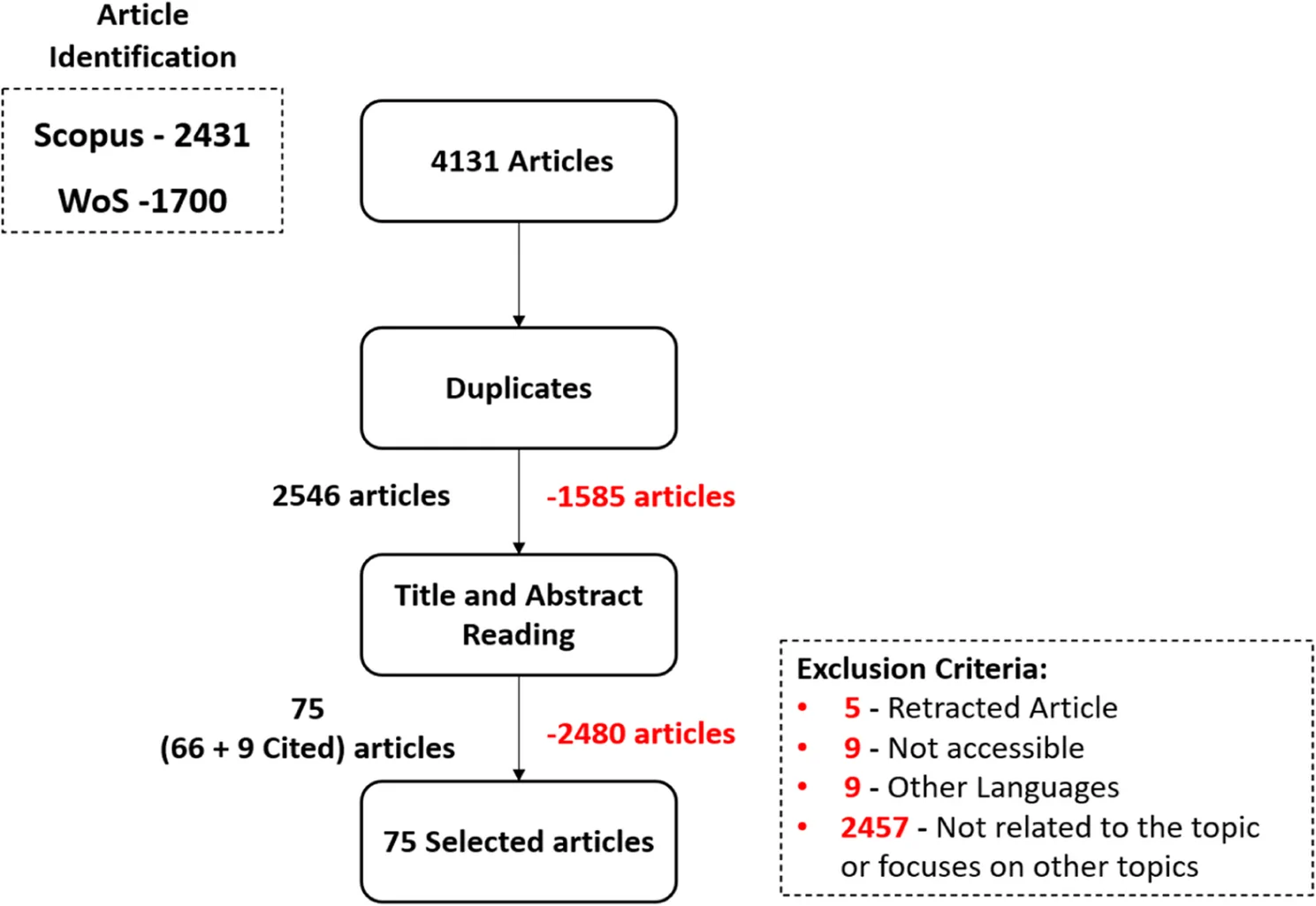
Summary of the protocol followed in the process of selection and review of articles

A total of 4131 articles were retrieved, and following the inclusion and exclusion criteria, 75 articles were selected for this review. The procedure followed during the selection of articles is given in Fig. 1.

**Table:** Summary of notations

*N*- Number of instances in total. *k*- Total number of classes $n_{i}$- Number of instances in$i^{th}$class $n_{min},n_{maj}$s- Number of instances in the minority and majority class, respectively $C_{i}$- Indicates$i^{th}$class	*max*- maximum *TP*- True Positive *TN*- True Negative *FP*- False Positive *FN*- False Negative

## Results

This section reviews research studies addressing multiclass imbalance. Section  3.1 focuses on approaches for handling multiclass classification problems. Section  3.2 examines class imbalance in multiclass classification problems and its measures. Section  3.3 outlines strategies to address these imbalances, and Section 3.4 details the evaluation metrics commonly applied in multiclass classification tasks.

Q1. How are multiclass classification problems commonly tackled?

### Handling multiclass classification

Initially, the multiclass problems were tackled by converting them into binary class problems using a binarization strategy [9]. One Versus One (OVO) and One Versus All (OVA) are the two commonly used binarization strategies. While comparing OVO and OVA under different aggregation strategies, such as voting, weighted voting, pairwise coupling, and others, OVO was found to be more effective for handling multiclass problems [9] and is further confirmed by Fernández et al. [10]. Further, Lango [1] employed OVO to deal with multi-class sentiment classification. Several strategies have been proposed to improve multiclass classification under class imbalance. Galar et al. [11] introduced the Distance-based Relative Competence Weighting OVO (DRCW-OVO) approach, which uses k-nearest neighbors(k-NN) to assess classifier competence and assign higher weights to classifiers associated with classes closer to the observation, mitigating misclassification in the standard OVO voting strategy. Zhang et al. [12] extended this method with the DRCW-ASEG approach, generating synthetic data to address class imbalance and incorporating it into the weighting process to enhance classifier competence. Shim [13] proposed the Relative Distribution OVO (RD-OVO) method, which incorporates relative distances to better represent class distributions and updates classifier weights accordingly. Zhang et al. [14] further improved these methods with the DRCW-FRkNN-OVO approach, which uses a fixed-radius nearest neighbor (NN) search to define the competence region within each class, providing more reliable competence estimation in skewed or sparsely distributed datasets. Apart from these proposed methods, Sen et al. [15] introduced Binarization with Boosting and Oversampling (BBO), an OVA-based framework that combines boosting and oversampling, highlighting the feasibility of combining binarization with ensemble and balancing techniques. Overall, these approaches demonstrate that incorporating class distribution, synthetic data, and local competence assessment can substantially improve multiclass classification performance in imbalanced settings. Table 1 summarizes the various decomposition strategies developed to address multiclass problems, highlighting each strategy’s prediction approach, strengths, and limitations.

**Table 1:** Summary table of different binarization strategies with strengths and limitations

Reference	Binarization Strategy	Classifier Prediction	Strengths	Limitations
Fernández et al. [9]; Galar et al. [10]	OVO	• Aggregates the votes or scores from all pairwise classifiers. • Uses the highest total score to predict the class label.	• Handles small class imbalances effectively. • Suitable for SVM implementations.	• Requires multiple classifiers • High computational complexity. • Aggregation of results is complex.
Fernández et al. [9]; Galar et al. [10]	OVA	• Each classifier distinguishes one class against all others. • Uses the highest confidence score to classify labels.	• Simple implementation with fewer classifiers. • Suitable for well-separated classes.	• Sensitive to class imbalance; can overfit on majority classes. • Moderate computational complexity (requires k classifiers)
Galar et al. [11]	DRCW-OVO	• Involves distance-based weighting of classifiers’ output by its competence score. • Uses maximum weighted score to predict class labels.	• Dynamic weighting improves classification competence. • Suitable for multi-class problems with noisy data.	• High computational complexity due to distance calculations and weighting mechanisms. • Requires fine-tuning for optimal performance. • Can struggle with biases in highly imbalanced datasets.
Zhang et al. [12]	DRCW-ASEG-OVO	• Uses competence weighting along with synthetic data generation • Uses maximum score after applying weighting and synthetic data to predict class labels	• Effectively balances class distribution. • Suitable for imbalanced and noisy datasets. • Achieves better results in complex datasets.	• High computational cost due to synthetic data generation and competence-based weighting.
Shim [13]	RD-OVO	• Adjusts class weights dynamically based on real-time data distribution and relative distances to predict the class label.	• Dynamically adjusts class weights, reducing imbalance bias.	• Assumes class distribution patterns. • Sensitive to noisy data. • Moderate complexity due to dynamic weight adjustments.
Zhang et al. [14]	DRCW-FRkNN-OVO	• Involves dynamic weight adjustment using fixed-radius kNN distances. • Uses weighted votes for predicting the class label	• Improves classifier competence by incorporating fixed-radius kNN. • Handles the sparse and imbalanced data.	• Requires careful tuning of the radius size. • Sensitive to noisy data. • High computational complexity due to kNN computations, especially for large datasets.

Q2. How can class imbalance be effectively measured and evaluated in multiclass classification problems?

### Class imbalance in multiclass classification and its measures

Many classification techniques are developed for balanced datasets, where each class contains approximately an equal number of instances, typically resulting in optimal accuracy. In imbalanced datasets where one class (the majority) has significantly more instances than others (the minority) these techniques tend to be biased toward the majority class, leading to reduced predictive performance. This problem is well-known in binary-class scenarios but becomes more severe in multiclass settings [16], where multiple minority and majority classes may coexist. Further, as the number of classes increases, the performance of classifiers tends to decline, with multi-majority classes having a greater impact than multi-minority classes [3–4] These minority and majority classes are typically identified using a threshold computed as the ratio of the size of the dataset to the number of classes.

$$\:Threshold=\:\frac{N}{k}$$

Any class $$\:{C}_{i}$$ with instances $$\:{n}_{i}>\raisebox{1ex}{$N$}\!\left/\:\!\raisebox{-1ex}{$k$}\right.$$ is considered as the majority class, while those with $$\:{n}_{i}<\raisebox{1ex}{$N$}\!\left/\:\!\raisebox{-1ex}{$k$}\right.$$ $$\:\forall\:\:i=\mathrm{1,2},\dots\:,\:k$$ is considered as the minority class [17, 18].

#### Imbalance measure

Class imbalance in binary classification problems is commonly quantified using the Imbalance Ratio (IR). When IR is defined as $\:\frac{{n}_{min}}{{n}_{maj}}\:$, IR ranges from 0 to 1, with 1 indicating balanced classes. When IR is defined as $\:\frac{{n}_{maj}}{{n}_{min}}$ values of 1 indicate balance, and values greater than 1 indicate imbalance [10, 19]. The class is said to be imbalanced if the ratio of minority to majority classes ranges from 1:4 to 1:100 and is extremely imbalanced if it ranges from 1:1000 to 1:5000 [16].Leevy et al. [5] describe extreme imbalance as a ratio from 100:1 to 10,000:1 and Hamid et al. [6] as 80:20, 90:10, or 95:5.

Ortigosa-Hernández et al. [20] observed that IR is useful for binary class problems but inadequate for multiclass problems and introduced the Imbalance Degree (ID), considering the sample size of each class. ID quantifies the imbalance based on (1) the distance between the actual and balanced class distribution, and (2) the number of minority classes. The drawback of ID is that the level of class imbalance measured is highly dependent on the number of minority classes. Although ID has a defined range of * [0,k)* bounded by the number of classes, Pirizadeh et al. [18] observed that it depends on the choice of distance measures and does not yield fixed range values.

To overcome the limitations of the ID, R. Zhu et al. [21] proposed the Likelihood Ratio Imbalance Degree (LRID), which uses the log-likelihood ratio to define the extent of class imbalance in both binary and multiclass problems. The LRID has notable limitations, including sensitivity to class sample sizes, which affects scalability, and the lack of a defined value range. In 2020, R. Zhu et al. [22] further noted that IR fails to adequately reflect imbalance across different dimensionalities and proposed an Adjusted Imbalance Ratio (AIR) with the inclusion of a dimensionality factor. The drawback of the AIR is that it has no definite range of values and is not suitable for multiclass problems.

Recently, Pirizadeh et al. [18] highlighted that existing imbalance measures do not meet essential conditions for both binary and multiclass datasets, leading to the proposal of a new measure called Imbalance Factor (IF). While IF primarily addresses inter-class imbalance, it does not consider intra-class imbalance or small disjuncts, which also affect classification performance. Zhao and Wu [23] introduced the Average Imbalance Ratio (AvIR), a measure suitable for quantifying class imbalance in multiclass datasets.

Table 2 summarizes above discussed imbalance measures outlining their properties such as the measure’s applicability to multiclass problems, its range, balance and imbalance values, key factors considered, and severity of imbalance defined. (✔ indicates method addresses the condition mentioned, while ✖ indicates it does not)

**Table 2:** Summary table of different imbalance measures with their properties

Reference	Imbalance Measure	Multi-class	Fixed Range	Balance	Imbalance	Key factors	Severity of imbalance
Fernández et al. [10]	IR	✖	✔ [0,1]	IR = 1	IR = 0	Sample size of classes	✔
Ortigosa-Hernández et al. [20]	ID	✔	✖	ID = 0	ID ≠ 0	Sample size of classes in both binary and multiclass	✖
Zhu et al. [21]	LRID	✔	✖	LRID = 0	Larger LRID	Log-likelihood ratio of imbalanced to exactly balanced data	✖
Zhu et al. [22]	AIR	✖	✖	Undefined	Larger AIR	Sample size of classes and number of variables	✖
Pirizadeh et al. [18]	IF	✔	✔ [0,1]	IF = 1	IF ≠ 1	Inter-class imbalance	✖
Zhao & Wu [23]	AvIR	✔	✖	-	-	IR of each class.	-

#### Impact of other data characteristics on multiclass imbalanced data

Classifier performance is affected not only by class imbalance but also by data difficulty factors associated with the minority class, including small or rare sub-concepts, class overlap, and outliers [3, 24]. Instances are generally categorized as safe or unsafe, with unsafe instances further classified as borderline, rare, or outliers [24, 25]. Multiclass imbalanced problems are more complex than binary cases, as a class can simultaneously act as a minority for some classes and a majority for others, creating ambiguity in resampling strategies [16, 24]. The concept of instance types has been extended to multiclass settings using a class similarity measure, where higher safe-level values indicate instances located in homogeneous regions of the same class, while the presence of other classes reduces the safe-level [24, 26]. Class overlap, particularly involving intermediate-sized and minority classes, further complicates the data and can obscure minority patterns, severely degrading classifier performance [3, 24]. Methods such as the multiclass ImGrid clustering algorithm have been developed to detect minority sub-concepts, overlapping regions, and outliers without extensive parameter tuning, thereby improving analysis and preprocessing of multiclass imbalanced datasets [24]. Additionally, class overlap in multiclass settings is quantified using Fisher’s discriminant ratio and the Augmented R value [3, 27].

Q3. How is class imbalance addressed in multiclass classification problems?

### Approach to handle the class imbalance: Balancing techniques

Class imbalance is typically addressed using balancing techniques that aim to balance the class distributions of minority and majority classes. There are three approaches to resolve class imbalance issues, namely, data-level, algorithm-level, and hybrid-level approaches [2].

#### Data-level approach

The data-level approach involves modifying the existing data to balance the class distribution using sampling techniques. This approach consists of three categories, namely under-sampling, oversampling, and hybrid sampling, which are discussed below.

##### Under-sampling

Under-sampling balances class distributions by removing instances from the majority class. Random under-sampling (RUS) is the most commonly used approach, which randomly eliminates samples from the majority class. A major limitation of most under-sampling methods is the potential loss of critical information, as important majority-class instances may be discarded, weakening the learning capability of classifiers for that class. Additionally, applying under-sampling alone is often ineffective for multiclass problems [28] and can adversely affect overall accuracy due to data removal [29]. To address these issues, Arafat et al. [30] proposed a support vector-based under-sampling technique that combines the decision points of majority-class support vectors with minority-class instances using Support Vector Machine (SVM) to balance the class distribution. Krawczyk et al. [31] introduced a two-phase under-sampling method, OCSVM-US, which selects a subset of support vectors, reducing computational cost, improving accuracy relative to oversampling methods, and enhancing robustness to noise and outliers. Additionally, a cluster-based under-sampling technique has been developed to handle multi-minority datasets with high IR [32].

##### Oversampling

Oversampling addresses class imbalance by increasing minority-class instances, either through duplication or synthetic generation, while preserving all majority class data. Random over sampling (ROS) duplicates minority instances but may cause overfitting. To mitigate this, synthetic oversampling methods such as SMOTE and its variants including Borderline SMOTE, Safe-Level SMOTE, and ADASYN have been developed, primarily for binary-class imbalance, to generate diverse minority instances and reduce learning bias.

For multiclass problems, several advanced oversampling techniques have been developed. Abdi & Hashemi [33] introduced Mahalanobis Distance-based Oversampling (MDO) which generates synthetic minority instances maintaining the same Mahalanobis distance from the class mean. This study compares the proposed method with ROS, SMOTE, ADASYN and Borderline SMOTE using OVA strategy. Kumari and Thakar [34] introduced Hellinger Distance-based Oversampling (HDO) to address class overlap and data skewness. In 2017, Zhu et al. [35] proposed a k-NN based Synthetic Minority Oversampling algorithm for multiclass imbalance problems (SMOM) to mitigate over-generalization issues observed in existing oversampling techniques. To overcome the limitations of MDO, such as over-generalization and its inability to handle mixed data types, Yang et al. [36] proposed Adaptive Mahalanobis Distance-based Oversampling (AMDO). Dentamaro et al. [37] introduced Less Important Components for Imbalanced Multiclass Classification (LICIC) to address both class imbalance and high-dimensional datasets. Krawczyk et al. [38] proposed Multiclass Radial-Based Oversampling (MC-RBO) to overcome the sensitivity of NN based oversampling to skewed distributions, class overlap, and small disjuncts by generating synthetic instances using radial basis functions. Two-tier iterative ensemble methods have also been developed to preserve the correlation structure of the original data by separating high and low informative samples using Z-scores, followed by ROS and constrained SMOTE to achieve balanced datasets [39, 40].

Liu et al. [41] developed a Modified Real-Value Negative Selection Detector-Based Oversampling Approach (MOA) to overcome the problems of overgeneralization and within-class imbalance commonly associated with SMOTE. Yao and Lin [19] noted that MDO and AMDO generate synthetic samples within a single ellipsoid, often causing class overlap. To address this, they proposed Evolutionary Mahalanobis Distance Oversampling (EMDO) method, which employs multiple ellipsoids to better capture the distribution and orientation of minority class samples. Li et al. [42] introduced SMOTE-IF, which first oversamples the minority class to handle severe imbalance and then applies an isolation forest to eliminate overlapping and noisy data. Tsai et al. [43] emphasized the importance of performing feature selection, particularly for ensemble-based models, prior to oversampling to enhance classification performance. Zhao and Wu [23] proposed Clustering-Based Oversampling Method (COM), which clusters minority instances based on structural similarity, assigns higher sampling weights to low-density clusters, and generates synthetic samples to alleviate intra-class imbalance and overgeneralization. A summary of these methods, including their imbalance measures, applicable data types, addressed issues, and limitations, is presented in Table 3.

**Table 3:** Summary of oversampling techniques for handling multiclass imbalance problems

Reference	Balancing technique	Data type	Imbalance measure	High imbalance	Classification Methods	Issue Addressed	Limitations
Abdi and Hashemi [33]	MDO	Continuous	IR	No	DT (C4.5), k-NN, RIPPER	• Class Overlap	• Time-consuming for high-dimensional data. • Applicable to continuous data only. • Use of Mahalanobis distance measure only
Kumari & Thakar [34]	HDO	Continuous	IR	No	k-NN, DT (C4.5)	• Class Overlap • Skewness	-
T. Zhu et al. [35]	SMOM	Continuous	IR	No	DT (C4.5), AdaBoost, Multilayer Perceptron (MLP), Naïve Bayes (NB)	• Overgeneralization	• Applicable to continuous data only.
Yang et al. [36]	AMDO	Both	IR	No	DT (C4.5)	• Overgeneralization • Mixed data type	• Performance of AMDO is limited to low-dimensional datasets.
Dentamaro et al. [37]	LICIC	Continuous	-	No	SVM, Random Forest (RF)	• Class imbalance • High Dimensionality	• Primarily applicable to high-dimensional data.
Sridhar & Kalaivani [39]	Two tier iterative ensemble	Not mentioned	Not used	No	RF	• Minority class recognition	-
Krawczyk et al. [38]	MC-RBO	Not mentioned	IR	No	DT (C5.0), MLP, k-NN, NB	• Class overlaps • Skewed data distributions • Small disjuncts	• Sensitive to noisy data
Liu et al. [41]	MOA	Continuous	IR	No	MLP, NB, SVM Logistic Regression	• Overgeneralization • Within-class imbalance	• Applicable to continuous data only • Setting of the parameters
Yao & Lin [19]	EMDO	Numerical	IR	Yes	DT (C4.5)	• Overgeneralization	-
A. Li et al. [42]	SMOTE-IF	Not mentioned	IR	Yes	k-NN	• Class Overlap • Noisy data	-
Zhao & Wu [23]	COM	Not mentioned	Average IR	No	DT, k-NN, MLP	• Intraclass imbalance • Overgeneralization	• High dimensionality

##### Hybrid sampling

Under-sampling techniques often result in information loss, whereas oversampling may cause overgeneralization. To mitigate these issues, hybrid sampling methods that combine both approaches are commonly employed. In hybrid sampling, oversampling is typically applied first to generate new instances, followed by undersampling to remove redundant ones. For multiclass imbalance problems, Prachuabsupakij and Soonthornphisaj [44] proposed KSMOTE, which integrates k-means clustering with SMOTE and RUS to balance class distributions; however, the method was evaluated only on continuous attributes and performs poorly with small datasets. Agrawal et al. [28] introduced the SMOTE and Clustered Undersampling Technique (SCUT) that combines SMOTE-based minority oversampling with cluster-based majority undersampling to address both between and within class imbalances. Janicka et al. [45] developed the Similarity Oversampling and Undersampling Preprocessing (SOUP) method, which resamples data based on instance difficulty derived from local class distributions, avoiding any binarization strategies. The Similarity-Based Hybrid Sampler (SHSampler) applies relative majority weakening undersampling and relative minority strengthening oversampling to handle complex interclass relationships and data difficulty [46]. Palli et al. [17] introduced Cluster-Based Hybrid Sampling for Imbalance Data (CBHSID), which uses affinity propagation clustering to create sub-clusters, removing samples far from centroids during undersampling and generating synthetic samples near centroids for oversampling, reducing information loss and overfitting.

Munguía Mondragón et al. [47] proposed a hybrid class balancing approach that combines density-based clustering with RUS and oversampling to address highly imbalanced multi-class problems, though it was evaluated only on image data. Existing multi-class balancing methods often focus on global IRs while neglecting factors such as class overlap and the decomposition of minority classes into sub-concepts. To overcome these limitations, Naglik and Lango [48] introduced GMMSampling, a resampling technique that employs a Gaussian Mixture Model (GMM) to generate synthetic minority instances. Table 4 provides a summary of these hybrid sampling methods, including the imbalance metrics used, applicable data types, the challenges tackled, and the limitations of each method. Several data-level approaches were introduced in the literature. Table 5 provides a summary of these methods based on their use of distance-based, cluster-based, or distribution-based approaches in the proposed method.

**Table 4:** Summary of hybrid sampling techniques for handling Multiclass imbalance problems:

Reference	Balancing technique	Data type	Imbalance measure	High imbalance	Classification Method	Issue Addressed	Limitations
Prachuabsupakij & Soonthornphisaj [44]	KSMOTE	Not mentioned	IR	Yes	• RF	• Multiclass imbalance	• Struggles with datasets containing small instances.
Agrawal et al. [28]	SCUT	Not mentioned	Not used	Yes	• DT (J48) • SVM • NB • IBk (NN)	• Both within-class and between-class imbalances.	• Choice of one over other oversampling method.
Janicka et al. [45]	SOUP	Not mentioned	Not used	No	• DT (J48) • PART Rule • k-NN	• Class Overlap	• Complexity of calculating safe levels and class similarities
Zheng et al. [46]	SHSampler	Not mentioned	ID	No	• DT • RF • NB	• Data difficulty factors • Mutual relationships	• Extreme data distribution
Palli et al. [17]	CBHSID	Not mentioned	IR	Yes	• SVM	• Overfitting • Information loss	• Not evaluated for the noisy data
Munguía Mondragón et al. [47]	• SCUT + DBS (SCUT + DBSCAN) • SCUT+HDBS (SCUT + HDBSCAN)	Image	IR	Yes	• Deep Neural Network	• Highly imbalanced multiclass data	• Limited evaluation on other data types.
Naglik and Lango [48]	GMM Sampling	Continuous	IR	No	• DT • NB • k-NN • RF • Gradient boosting trees	• Class Overlap	• Applicable to continuous data only. • Computational complexity due to an increase in dimensionality

**Table 5:** Summary of data-level approach for handling multiclass imbalance problems:

Balancing approach	Balancing technique	Reference	Distance-based	Cluster-based	Distribution based
Oversampling	MDO	Abdi and Hashemi [33]	✔	✖	✖
	HDO	Kumari & Thakar [34]	✔	✖	✖
	SMOM	T. Zhu et al. [35]	✔	✖	✖
	AMDO	Yang et al. [36]	✔	✖	✖
	LICIC	Dentamaro et al. [37]	✔	✖	✖
	MC-RBO	Krawczyk et al. [38]	✖	✔	✔
	MOA	Liu et al. [41]	✔	✖	✖
	EMDO	Yao & Lin [19]	✔	✖	✖
	SMOTE -IF	A. Li et al. [42]	✖	✖	✖
	COM	Zhao & Wu [23]	✔	✔	✖
Hybrid Sampling	KSMOTE	Prachuabsupakij & Soonthornphisaj [44]	✔	✔	✖
	SCUT	Agrawal et al. [28]	✔	✔	✔
	SOUP	Janicka et al. [45]	✔	✖	✖
	SHSampler	Zheng et al. [46]	✔	✖	✖
	CBHSID	Palli et al. [17]	✔	✔	✖
	SCUT + DBS and SCUT+HDBS	Munguía Mondragón et al. [47]	✔	✔	✖
	GMM Sampling	Naglik and Lango [48]	✖	✖	✔

#### Algorithm level approach

The algorithmic level approach focuses on adapting the classification algorithm to effectively handle the class imbalance problems [26]. Hoens et al. [49] introduced the Multi-Class Hellinger Distance Decision Tree (MC-HDDT), which adopts Hellinger distance as a splitting criterion to improve decision tree (DT) performance on multiclass imbalanced data. A One-Against-All Hellinger distance (OAHD), extension of the Hellinger distance refines the splitting rule of DT to enhance discrimination among multiple classes, as demonstrated by Dong et al. [50]. Sridhar and Anusuya [51] developed a Dual DT framework which applies DTs in two stages: the first tree groups data using PCA and assigns sample weights to create an induced dataset, and the second tree uses this dataset to predict class labels. The algorithm-level approach leads to two more approaches: cost-sensitive learning and ensemble-level approaches.

##### Cost-sensitive learning

Resampling techniques often suffer from information loss and overfitting due to alterations in the original data distribution. Cost-sensitive learning provides a more robust alternative by assigning different misclassification costs during training while preserving the data structure [52]. This approach assigns higher costs to minority class misclassifications to improve balance [19, 53]. To address classifier bias and noise sensitivity, Phoungphol et al. [54] proposed a cost-sensitive multiclass SVM with ramp loss (Ramp-MCSVM). Similarly, Nababan et al. [55] introduced a cost-sensitive k-NN based on the MetaCost framework, and Febriantono et al. [56] developed a cost-sensitive C5.0 DT that prioritizes minority classes by weighting classification errors accordingly.

##### Ensemble methods

Ensemble approaches combine techniques such as boosting, bagging, and stacking with data-level or cost-sensitive strategies to handle class imbalance [19, 57]. Azari et al. [57] introduced MILES, which integrates ensemble learning with cluster-based selective sampling. Lango and Stefanowski [58] proposed Multiclass Roughly Balanced Bagging (MRBBag) to manage multiple minority classes and complex data difficulty factors, and later developed SOUP-Bagging, an ensemble version of SOUP that applies bagging during training [26]. In boosting-based approaches, Wang and Yao [4] introduced AdaBoost.NC, which mitigates the impact of multi-minority and multi-majority classes without requiring OVA decomposition, while Abdi and Hashemi [59] proposed MDOBoost, combining Mahalanobis distance-based oversampling with boosting. Rodríguez et al. [60] extended the Random Balance strategy to multiclass problems through MultiRandBal, OVO-RandBal, and OVA-RandBal, with OVA showing better performance. More recently, So and Valdez [61] developed SAMME.C2, integrating ensemble and cost-sensitive learning to address extreme imbalance. Table 6 provides the summary of these methods.

**Table 6:** Summary of algorithmic level approaches for handling multiclass imbalance problems:

Reference	Algorithmic approach	Data type	Imbalance measure	High imbalance	Classification method	Issue Addressed	Limitations
Algorithmic approach							
Hoens et al. [49]	MC-HDDT	Not mentioned	IR	Yes	MC-HDDT	Multiclass imbalance	**-**
Dong et al. [50]	OAHD DT	Not mentioned	IR	No	OAHD DT	Skewed distribution of multiclass data	**-**
Sridhar & Anusuya [51]	Duo Decision Tree	Not mentioned	Not Used	No	DT	Multi-majority and multi minority	Minority class with extremely low count
Cost-Sensitive approach							
Phoungphol et al. [54]	Ramp-MCSVM	Not mentioned	IR	Yes	SVM	Noise Highly imbalanced data	**-**
Febriantono et al. [56]	Cost-Sensitive DT (C5.0)	Not mentioned	Not used	No	DT (C5.0)	Multiclass imbalance	Use of accuracy as the performance measure
Nababan et al. [55]	Cost -Sensitive k-NN	Not mentioned	Not used	No	k-NN	Multiclass imbalance	-
Ensemble approach							
Azari et al. [57]	MILES	Not mentioned	Not used	No	DT (C4.5)	Multiclass imbalance	-
Lango & Stefanowski [58]	MRBBag	Not mentioned	IR	Yes	DT (J4.8)	Multiclass imbalance without binary decomposition	Use of a single classifier Struggles to classify unsafe majority examples
Lango & Stefanowski [26]	SOUP Bagging	Not mentioned	Not used	No	DT (J4.8)	Interrelation between multiple classes.	The complexity of calculating safe levels and class similarities
Shuo Wang & Xin Yao [4]	AdaBoost.NC	Not mentioned	Not used	No	DT (C4.5)	Multi-minority and multi-majority classes without decomposition	Predefined parameter of the proposed model.
Abdi & Hashemi [59]	MDOBoost	Not mentioned	IR	No	DT (C4.5, C4.5UP) RIPPER, NB	Multiclass imbalance	-
Rodríguez et al. [60]	MultiRandBal	Both	IR	No	DT	Multiclass imbalance	Considered DT as a base classifier and not evaluated for other base classifiers.
So & Valdez [61]	SAMME C2	Both	Class Proportion	Yes	Decision Stump	Extremely imbalanced data	-

#### Hybrid level approach

Hybrid approaches combine data-level and algorithm-level techniques to effectively handle multiclass imbalance. García et al. [62] proposed Dynamic Ensemble Selection for Multiclass Imbalanced datasets (DES-MI), which integrates ROS, RUS, and SMOTE within a random balance framework to achieve balanced class distributions. Hartono et al. [63] introduced the Hybrid Approach Redefinition–Multiclass Imbalance (HAR-MI), combining RUS and SMOTEBoost with Dynamic Ensemble Selection (DES). To overcome HAR-MI’s overfitting issues due to oversampling, Hartono et al. [64] later incorporated a Complexity-based Oversampling Technique (COSTE), which focuses on complex instances but remains sensitive to high IRs. To further minimize noise and overlap, a hybrid method called Minimizing Overlapping Selection under Hybrid Sampling (MOSHS) was introduced [65].

Ongko and Hartono [66] enhanced hybrid learning by integrating resampling with feature selection at both preprocessing and processing stages, while another hybrid approach with a distance feature minimized class overlap by identifying safe regions for generating synthetic instances [27]. Sridhar and Anusuya [67] addressed the limitations of binarization-based methods by proposing a Two-tier Iterative Ensemble Sampling and Duo Decision Tree framework, effectively managing extreme multiclass imbalance involving both multi-majority and multi-minority classes. The summary of these hybrid approaches was given in the Table 7.

**Table 7:** Summary of hybrid approaches for handling multiclass imbalance problems:

Reference	Hybrid approach	Data-Level approach	Algorithm-level approach	Data type	Imbalance measure	High imbalance	Issue Addressed	Limitations
García et al. [62]	DES- MI	SMOTE, RUS	DES	Both	IR	No	• Multiclass imbalance	• Noise and overfitting due to SMOTE
Hartono, Risyani, et al. [63]	HAR-MI	Random Balance Ensembles, SMOTEBoost	DES	Not mentioned	IR	No	• Multiclass imbalance	• Choice of sampling method
Hartono, Lestari, et al. [64]	HAR-MI with COSTE	COSTE (Complexity-based Oversampling)	DES	Not mentioned	IR	Yes	• Overfitting	• Large number of features • High IR
Hartono & Ongko [65]	HAR-MI with Hybrid Sampling	Hybrid Sampling (SMOTE and RUS)	Classifier ensemble	Not mentioned	IR	Yes	• Overfitting • Class overlap	• High IR with class overlap
Ongko & Hartono [66]	HAR-MI with resampling and feature selection	MOSS (Minimizing Overlapping Selection under SMOTE), MC-CCR (Multi-class Combined Cleaning and Resampling)	DES Random Balance Ensemble (RBE)	Not mentioned	IR	Yes	• Class overlap • Noise	• Large number of attributes
Hartono & Ongko [27]	Hybrid Approach with Distance Feature	Distance-based Sampling	Ensemble of classifiers	Not mentioned	IR	No	• Class overlap • Class imbalance	-
Sridhar & Anusuya [67]	Two-tier iterative ensemble sampling and Duo Decision Tree Approach	Iterative Ensemble Sampling	Duo Decision Tree	Not mentioned	Not used	Yes	• Class overlap • Overfitting • Low recognition of minority class • Multi-majority and multi-minority	• Computational complexity

Q4. How is the performance of classifiers measured in the case of multiclass settings?

### Evaluation metrics for multiclass imbalanced data

Performance measures assess how well a classification model predicts the correct class for unknown instances. These metrics are generally categorized as overall or class-specific (single-class) performance measures [2]. Overall performance metrics such as accuracy and the Area Under the ROC Curve (AU-ROC) provide a single-value summary of model performance across all classes. While these metrics are commonly used in binary classification, they often fail to capture model performance effectively in imbalanced datasets. In contrast, class-specific measures like sensitivity and specificity evaluate how well the model classifies individual classes, offering deeper insights in the presence of imbalance. Although numerous metrics exist for binary classification, multiclass problems require specialized measures to capture performance differences across multiple classes.

For multiclass settings, Sun et al. [68] proposed the Geometric Mean (G-Mean) of Recall to evaluate performance across individual classes, while Mosley [69] introduced Macro Average Precision (MAP) and Macro Average Recall (MAR), which summarize per-class results into overall metrics suitable for multiclass imbalance analysis. Hand & Till [70] introduced Multiclass Area Under the Curve (MAUC) by pairwise averaging AU-ROC, generalizing the ROC to multiclass problems. Mosley [69] introduced overall accuracy, F-score and Class Balance Accuracy (CBA) specifically for imbalanced multiclass problems. Tanha et al. [2] extended the Matthews Correlation Coefficient (MCC) metric to the multiclass problems by introducing Multiclass MCC (MMCC) using the OVO decomposition strategy inspired by MAUC.

**Fig. 2:**
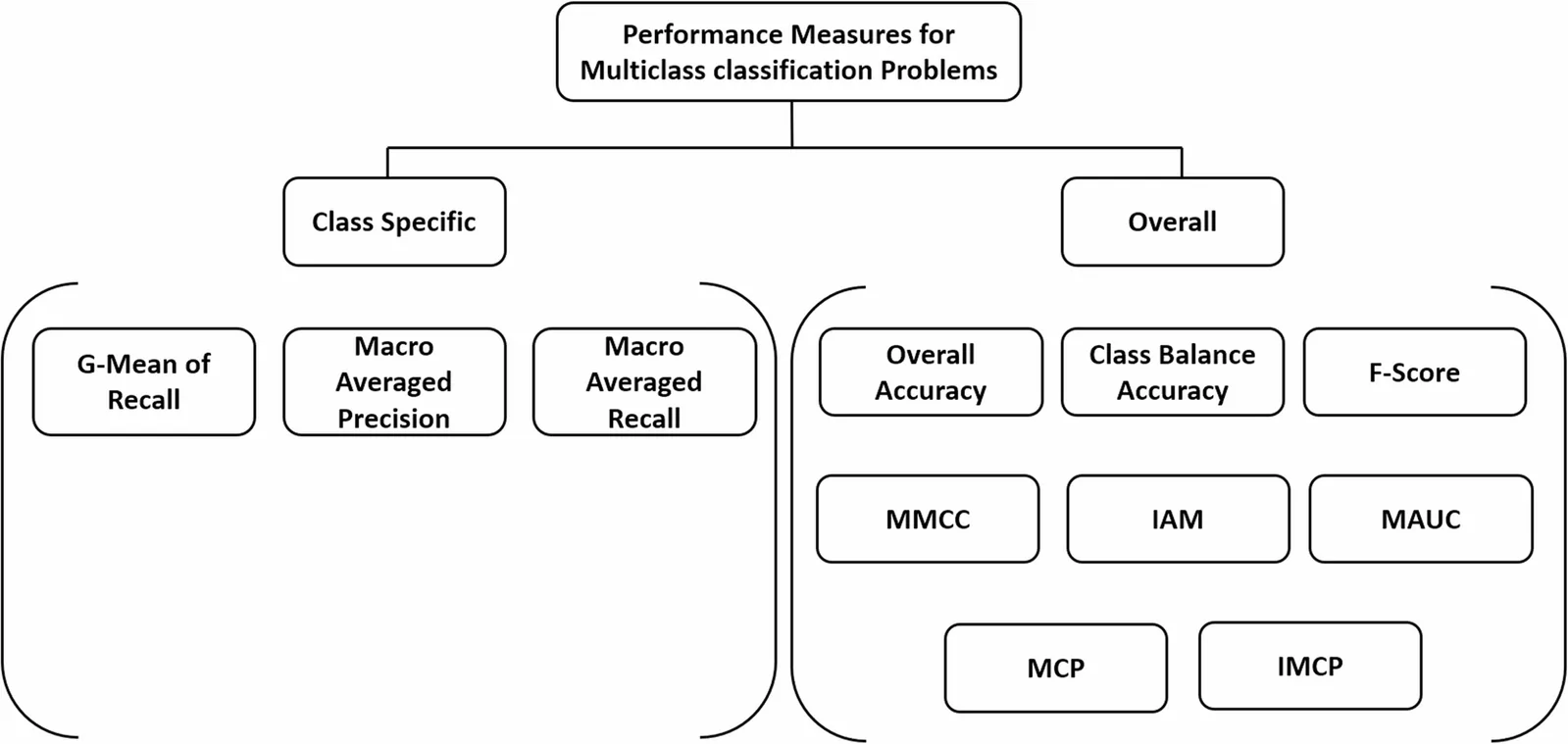
Categorization of performance measures for multiclass classification problems

To overcome the limitations of class imbalance in existing multiclass performance metrics, Mortaz [71] introduced the Imbalance Accuracy Metric (IAM) for more robust model evaluation and selection.Aguilar-Ruiz & Michalak [72] introduced a Multiclass Classification Performance (MCP) curve based on the Hellinger distance. Recently, Aguilar-Ruiz et al. [73] proposed the Imbalanced Multiclass Classification Performance (IMCP) Curve and the area under the IMCP curve to assess classification quality in imbalanced multiclass scenarios. The categorisation of these proposed measures into class-specific and overall performance measures is given in Fig. 2. A summary of the performance measures used in the case of multiclass problems is given in Table 8.

**Table 8:** Summary of the performance measures in case of multiclass problems

References	Performance measure	Measure Type	Class Imbalance	Formula
Hand & Till [70]	Multiclass Area Under the Curve (MAUC)	Overall performance	No	$MAUC=\frac{2}{{k\left(k-1\right)}}\sum\limits_{i<j}\hat{A}\left(i.j\right)$
Sun et al. [68]	G-Mean of Recall	Class-specific performance	Yes	$\:G-Mean=\:{\left(\prod\:_{i=1}^{k}{R}_{i}\right)}^{1/k}$, where$\:{R}_{i}$is the recall value of$\:{i}^{th}$class
Mosley ; Mortaz [69, 71]	Macro Average Precision (MAP)	Class-specific performance	No	$\:MAP=\:\frac{1}{k}\sum\:_{i=1}^{k}\frac{{C}_{ii}}{{C}_{.i}}\:,\:\mathrm{w}\mathrm{h}\mathrm{e}\mathrm{r}\mathrm{e}\:{C}_{.i}=\sum\:_{j=1}^{k}{C}_{ji}\:\:\forall\:i\in\:\{1,\dots\:,k\}$
Mosley; Mortaz [69, 71]	Macro Average Recall (MAR)	Class-specific performance	No	$\:MAP=\:\frac{1}{k}\sum\:_{i=1}^{k}\frac{{C}_{ii}}{{C}_{i.}}\:\:,\:\mathrm{w}\mathrm{h}\mathrm{e}\mathrm{r}\mathrm{e}\:{C}_{i.}=\sum\:_{j=1}^{k}{C}_{ij}\:\:\forall\:i\in\:\{1,\dots\:,k\}$
Mosley; Mortaz [69, 71]	Overall Accuracy	Overall performance	No	$\:ACC=\:\frac{\sum\:_{i}{C}_{ii}}{\sum\:_{ij}{C}_{ij}}$
Mosley; Mortaz [69, 71]	F-score (Macro)	Overall performance	No	$\:F=\frac{1}{k}\:\sum\limits_{i=1}^{k}\frac{2.\frac{{C}_{ii}}{{C}_{.i}}\frac{{C}_{ii}}{{C}_{i.}}}{\frac{{C}_{ii}}{{C}_{.i}}+\frac{{C}_{ii}}{{C}_{i.}}}$
Mosley; Mortaz [69, 71]	Class Balance Accuracy (CBA)	Overall performance	Yes	$\:CBA=\frac{1}{k}\:\sum\limits_{i=1}^{k}\frac{{C}_{ii}}{\mathrm{m}\mathrm{a}\mathrm{x}({C}_{.i},{C}_{i.})}$
Tanha et al. [2]	Multi-Class Matthews Correlation Coefficient (MMCC)	Overall performance	Yes	$MMCC=\frac{2}{k\left(k-1\right)}\sum\limits_{i<j}MCC\left(i.j\right)$ where$\:MCC=\frac{TP\times\:TN-FP\times\:FN}{\sqrt{(TP+FP)(TP+FN)(TN+FP)(TN+FN)}}$
Mortaz [71]	Imbalance Accuracy Metric (IAM)	Overall performance	Yes	$\:IAM=\frac{1}{k}\sum\limits_{i=1}^{k}\frac{{C}_{ii}-\mathrm{m}\mathrm{a}\mathrm{x}\mathrm{(}\sum\:_{j\ne\:i}^{k}{C}_{ij},\sum\:_{j\ne\:i}^{k}{C}_{ji}}{\mathrm{m}\mathrm{a}\mathrm{x}\left({C}_{.i},{C}_{i.}\right)}$
Aguilar-Ruiz & Michalak [72]	Multi-class Classification Performance (MCP)	Overall Performance	No	$\:AU\left(MCP\right)=\frac{1}{n-1}\left(\sum\limits_{i=1}^{n}{\varnothing\:}^{{\prime\:}}\left(i\right)-\left(\frac{{\varnothing\:}^{{\prime\:}}\left(1\right)+{\varnothing\:}^{{\prime\:}}\left(n\right)}{2}\right)\right)$
Aguilar-Ruiz et al. [73]	Imbalanced Multi-class Classification Performance (IMCP)	Overall performance	Yes	$\:AU\left(IMCP\right)=\:\frac{{{\delta\:}_{1}}^{{\prime\:}}{{\varnothing\:}_{1}}^{{\prime\:}}}{2}+\sum\limits_{i=1}^{n-1}\left(\frac{{{\delta\:}_{i}}^{{\prime\:}}+{{\delta\:}_{i+1}}^{{\prime\:}}}{2}\right)\left(\frac{{{\varnothing\:}_{i}}^{{\prime\:}}+{{\varnothing\:}_{i+1}}^{{\prime\:}}}{2}\right)+\frac{{{\delta\:}_{n}}^{{\prime\:}}{{\varnothing\:}_{n}}^{{\prime\:}}}{2}$

## Discussion

Handling multiclass classification problems introduces challenges that are more complex than those encountered in binary classification. Common binarization strategies, such as OVO and OVA, suffer from several limitations. A major concern is their susceptibility to class imbalance, which often results in biased predictions toward majority classes when some classes contain substantially fewer samples. In addition, these strategies require training multiple classifiers, leading to increased computational complexity. OVA and OVO exhibit important trade-offs in memory usage, training time, prediction complexity, and handling of class imbalance. OVA is generally more scalable for very large datasets because it requires training only * k* classifiers, leading to lower memory consumption and reduced computational cost. In contrast, OVO requires $\:k(k-1)/2\:$classifiers, substantially increasing computational burden, storage requirements, and prediction time as the number of classes increases. However, OVO often achieves better class discrimination and improved classification performance, particularly when combined with preprocessing or cost-sensitive learning techniques. Consequently, OVA is typically preferred for large-scale and high-dimensional applications where computational efficiency is important, whereas OVO is more suitable when predictive performance and finer class separation are prioritized. Thus, the selection of OVA, OVO or related approaches should depend on dataset size, number of classes, and available computational resources.

The literature identifies two imbalance measures for binary classification and three for multiclass classification. These measures are defined based on various factors, including sample size, number of variables, log-likelihood ratios, and interclass imbalance. Among them, IR is the only measure that explicitly quantifies the severity of imbalance in binary classification problems. Other measures, such as ID and IF provide a broader perspective for multiclass imbalance but do not quantify the severity of imbalance in a fixed or absolute manner.

The traditional IR is mainly designed for binary classification problems and offers straightforward interpretation due to its simple formulation and fixed range. However, its applicability becomes limited in multiclass settings. To address this limitation, measures such as ID, LRID, IF, and AvIR have been proposed for multiclass imbalance assessment. Among these, ID and LRID incorporate distributional and distance-based characteristics. However, their dependence on class distribution, distance metrics, and sample size reduces stability. In contrast, IF provides a bounded and relatively more stable measure, improving comparability across different multiclass scenarios, although it may not fully reflect the true severity of imbalance. From an interpretability perspective, IR remains the most transparent and computationally simple measure, whereas ID and LRID involve more complex mathematical formulations that may limit their practical interpretability. AvIR improves multiclass applicability by averaging pairwise imbalance information but may oversimplify local imbalance structures. Overall, the comparative analysis indicates that no single measure simultaneously achieves broad applicability, stability, and interpretability, highlighting the continuing need for more standardized, scalable, and practically interpretable multiclass imbalance measures.

In case of data-level approaches for multiclass imbalance, most of these methods focus on oversampling and hybrid sampling techniques, while comparatively fewer studies emphasize undersampling. Many of these methods are extensions of techniques originally developed for binary classification problems [38]. Broadly, data-level approaches are based on distance-based, clustering-based, and distribution-based strategies. Distance measures play a crucial role in generating synthetic data for minority classes, typically using nearest-neighbor concepts. Commonly used distance measures include the L1 norm [38], Heterogeneous Value Difference Metric (HVDM) [36, 45], Mahalanobis distance [19, 33], Euclidean distance [28, 41, 44], and Standardized Euclidean distance [35]. Clustering-based techniques, such as k-means and density-based clustering, have also been shown to be effective in handling multiclass imbalance. These techniques are generally applied in two ways. In the first approach, data are partitioned into clusters, after which balancing techniques such as oversampling are applied within each cluster to generate synthetic minority class instances [44]. In the second approach, clustering itself acts as a balancing mechanism, for example by undersampling majority class instances to achieve a more balanced class distribution [28, 47].

Algorithm-level approaches primarily modify the internal learning mechanisms of classifiers, with DTs being the most commonly adapted models. Cost-sensitive learning methods are predominantly applied to SVMs and DT models, where higher misclassification costs are assigned to minority classes. Ensemble-based approaches frequently integrate data-level strategies to mitigate class imbalance and enhance predictive performance. Overall, DTs remain the most widely employed classifiers across these algorithm-level and ensemble-based techniques.

Only a limited number of hybrid approaches have been proposed in the literature to address multiclass imbalance problems. These methods typically combine the data-level approaches such as RUS, SMOTE, or hybrid sampling techniques with algorithm-level strategies such as DES and DTs. The primary issues addressed by these approaches include class overlap and overfitting. However, high levels of class imbalance remain a major limitation of existing hybrid approaches [64, 65]. Moreover, most hybrid methods do not incorporate decomposition strategies, with the notable exception of HAR-MI with COSTE [64]. Recently, several hybrid-level approaches were introduced that focus on addressing class overlap and overfitting in the data however, high-class imbalance is still a challenge for these proposed methods. Several proposed methods also considered feature selection methods within them [27, 58, 66] and a new feature selection approach was also introduced, specifically for multiclass imbalance problems [74]. Oversampling and algorithmic approaches are widely used in the literature, as also observed by S. Li et al. [75].

Recent research trends reveal a significant shift from traditional binarization-based approaches towards more advanced and adaptive strategies for handling multiclass imbalance problems. Specifically, there is a transition from traditional binarization strategies to newer binarization approaches that incorporate dynamic weighting strategies and underlying class distribution characteristics to account more effectively for class imbalance. Recent developments also incorporate likelihood-based and class-distribution-based imbalance measures that account for class-specific sample sizes. Furthermore, increasing attention has been given to data difficulty factors such as class overlap, noisy instances, minority sub-concepts, and outliers, as these factors substantially affect classification performance beyond imbalance alone. At the data-level, advanced resampling techniques including distance-based, distribution-based, and clustering-based approaches have gained prominence, while algorithm-level and hybrid methods have been specifically developed to improve robustness in multiclass settings. In addition, the integration of ensemble learning with sampling and weighting strategies has emerged as an important direction for enhancing model stability and generalizability. Finally, recent research also highlights development of evaluation metrics specifically designed for imbalanced multiclass data, reflecting a growing emphasis on fairer and more reliable performance assessment. Overall, while significant progress has been made in addressing multiclass imbalance, existing methods still face limitations in handling severe imbalance, scalability, and class decomposition in highly imbalanced multiclass classification problems.

## Conclusion

Handling class imbalance in multiclass classification tasks is a critical challenge, and several methods have been introduced in the literature to address it. This review provides a comprehensive overview of methods that address the multiclass imbalance problems. A total of 75 articles published in the domain of multiclass imbalance were reviewed and summarized.

In conclusion, despite the introduction of several new measures for addressing multiclass imbalance, the IR remains the most widely used metric for determining the severity of imbalance (see Tables 3 and 4). Furthermore, various binarization strategies and their variants have been employed to address multiclass problems, with each offering distinct advantages and limitations. Most balancing techniques developed to mitigate class imbalance rely on distance-based, cluster-based, or distribution-based approaches, reflecting the diversity of methodological perspectives in the field. In addition, numerous performance evaluation metrics for multiclass problems have been investigated, underscoring the importance of selecting appropriate measures for accurate and fair assessment of classifier performance. Collectively, these insights highlight both the progress achieved and the continuing challenges in effectively managing multiclass imbalance.

In summary, this review contributes to the field of multiclass imbalance by providing a comprehensive assessment of existing methods, thereby offering valuable guidelines for researchers. Based on the review, future research should focus on following key areas to address the challenges in managing multiclass imbalance.

- Comparison of various decomposition strategies in the case of a larger number of classes and high-dimensional data using different aggregation strategies. Also, evaluate the computational complexity in such situations.
- Exploring the existing multiclass imbalance measures to define the severity of imbalance in the dataset.
- Incorporation of the algorithmic approach for handling class imbalance in classification models other than k-NN, SVM and DT.
- Development of balancing techniques that can handle multiple data difficulty factors.
- Development of the performance measures that consider the data difficulty factors such as class overlap, small disjuncts during computation.

In multiclass classification problems, various decomposition strategies, classification algorithms, and performance metrics have been proposed to address class imbalance. However, incorporating imbalance handling strategies such as class weight adjustments, separately at all three stages: decomposition, model training, and performance evaluation may introduce bias, as these adjustments are applied repeatedly. Therefore, future research should focus on assess the impact of applying these methods simultaneously on the performance of model to develop guidelines for the appropriate and balanced integration of such methods in multiclass classification tasks.

### Key factors to be considered when dealing with multiclass imbalance problems

The choice of different classification methods, balancing techniques, and performance measures in the context of imbalanced data depends on various data characteristics such as the number of features, dimensionality, sample size, data distribution, number of classes, and the severity of imbalance. However, several studies fail to mention these data characteristics, making it difficult to assess the feasibility and generalizability of the proposed methods across different data characteristics. Therefore, it is crucial to highlight the key factors that should be reported when dealing with imbalanced multiclass data. These factors help readers to gain a clear understanding of the scope and applicability of the proposed method. A summary of these key factors identified in the reviewed articles is provided in the table below, which future studies in this domain are recommended to consider.

**Table:** 

Factors		Details
Number of Samples	:	Total number of instances in the dataset.
Number of Variables	:	Count of all features (predictor variables) considered.
Response Variable	:	Type of response variable: Nominal or Ordinal
Number of Classes	:	Number of distinct classes in the target variable
Predictor Variables	:	Number of continuous and discrete (categorical) predictor variables
Distribution of Predictor Variables	:	Examination of how predictor variables are distributed (e.g., normal, skewed).
Imbalance Measure Used	:	Metrics used to quantify class imbalance and its severity
Missing Values Handling	:	Identification of missing values and imputation techniques used.
Classification Methods	:	Types of classification models used, such as DTs, SVM, neural networks, and their applicability to multiclass problems, their parameters considered.
Balancing Technique Used	:	Approaches to handle imbalance: Data-level (resampling), Algorithm level (cost-sensitive learning), Hybrid methods.
Distance measures used	:	Distance measures used in the classification method, as well as the balancing technique
Evaluation metrics	:	Use of suitable performance measures.
Decomposition strategy	:	Use of decomposition strategy in the classification method, balancing technique and evaluation metrics.

## Data Availability

No datasets were generated or analysed during the current study.

## Abbreviations

AMDO: Adaptive Mahalanobis Distance-based Over-sampling
CBA: Class Balance Accuracy
CBHSID: Cluster-based Hybrid Sampling for Imbalance Data
COM: Cluster-based Over-sampling Method
COSTE: Complexity-based Oversampling
DES: Dynamic Ensemble Selection
DES- MI: Dynamic Ensemble Selection for Multi-class Imbalanced Datasets
DRCW-ASEG-OVO: Distance-based Relative Competence Weighting with Adaptive Synthetic Example Generation -OVO
DRCW-FRkNN-OVO: Distance-based Relative Competence Weighting Fixed Radius k-Nearest Neighbour-OVO
DRCW-OVO: Distance-based Relative Competence Weighting-OVO
DT: Decision Tree
EMDO: Evolutionary Mahalanobis Distance Oversampling
GMM: Gaussian Mixture Model
HAR-MI: Hybrid Approach Redefinition-Multiclass Imbalance
HDO: Hellinger Distance-based Oversampling
IAM: Imbalance Accuracy Metric
ID: Imbalance Degree
IF: Imbalance Factor
IMCP: Imbalanced Multi-class Classification Performance
IR: Imbalance Ratio
k-NN: k-Nearest Neighbours
LICIC: Less Important Components for Imbalanced Multiclass Classification
LRID: Likelihood Ratio Imbalance Degree
MAP: Macro Average Precision
MAR: Macro Average Recall
MAUC: Multi-class Area Under the Curve
MCC: Matthew’s correlation coefficient
MCP: Multi-class Classification Performance
MC-RBO: Multiclass Radial-Based Oversampling
MDO: Mahalanobis Distance-based Oversampling
MLP: Multi-Layer Perceptron
MMCC: Multiclass Matthew’s Correlation Coefficient
MOA: Modified real-value negative selection detector-based Oversampling Approach
MRBBag: Multiclass Roughly Balanced Bagging
OVA: One Versus All
OVO: One Versus One
RD-OVO: Relative Distribution of Class OVO
RIPPER: Repeated Incremental Pruning to Produce Error Reduction
ROS: Random Oversampling
RUS: Random Undersampling
SCUT: SMOTE & Clustered Undersampling Technique
SHSampler: Similarity-based hybrid sampling
SMOM: Synthetic minority oversampling technique for multiclass imbalance problems
SMOTE: Synthetic Minority Over-sampling Technique
SMOTE-IF: SMOTE-Isolation Forest
SOUP: Similarity Oversampling and Undersampling Preprocessing
SVM: Support Vector Machine
OCSVM-US: One-Class Support Vector Machine-Undersampling

## References

[1] Lango M. Tackling the Problem of Class Imbalance in Multi-class Sentiment Classification: An Experimental Study. Found Comput Decis Sci. 2019;44(2):151–78.

[2] Tanha J, Abdi Y, Samadi N, Razzaghi N, Asadpour M. Boosting methods for multi-class imbalanced data classification: an experimental review. J Big Data. 2020;7(1):70.

[3] Lango M, Stefanowski J. What makes multi-class imbalanced problems difficult? An experimental study. Expert Syst Appl. 2022;199:116962.

[4] Wang S, Yao X. Multiclass Imbalance Problems: Analysis and Potential Solutions. IEEE Trans Syst Man Cybernetics Part B (Cybernetics). 2012;42(4):1119–30. 10.1109/TSMCB.2012.2187280 22438514

[5] Leevy JL, Khoshgoftaar TM, Bauder RA, Seliya N. A survey on addressing high-class imbalance in big data. J Big Data. 2018;5(1):42.

[6] Hamid MHA, Yusoff M, Mohamed A. Survey on Highly Imbalanced Multi-class Data. Int J Adv Comput Sci Appl. 2022;13(6):211-29.

[7] Patel V, Bhavsar H. An Empirical Study of Multi-class Imbalance Learning Algorithms. ICT Systems and Sustainability Lecture Notes in Networks and Systems. Singapore: Springer; 2023. pp. 161–74.

[8] Wang Y, Rosli MM, Musa N, Li F. Multi-Class Imbalanced Data Classification: A Systematic Mapping Study. Eng Technol Appl Sci Res. 2024;14(3):14183–90.

[9] Galar M, Fernández A, Barrenechea E, Bustince H, Herrera F. An overview of ensemble methods for binary classifiers in multi-class problems: Experimental study on one-vs-one and one-vs-all schemes. Pattern Recognit. 2011;44(8):1761–76.

[10] Fernández A, López V, Galar M, del Jesus MJ, Herrera F. Analysing the classification of imbalanced data-sets with multiple classes: Binarization techniques and ad-hoc approaches. Knowl Based Syst. 2013;42:97–110.

[11] Galar M, Fernández A, Barrenechea E, Herrera F. DRCW-OVO: Distance-based relative competence weighting combination for One-vs-One strategy in multi-class problems. Pattern Recognit. 2015;48(1):28–42.

[12] Zhang ZL, Luo XG, González S, García S, Herrera F. DRCW-ASEG: One-versus-One distance-based relative competence weighting with adaptive synthetic example generation for multi-class imbalanced datasets. Neurocomputing. 2018;285:176–87.

[13] Shim SO. Multi-Class Classification based on Relative Distribution of Class. In:, Sciences I. (ICCIS). IEEE; 2020. pp. 1–4.

[14] Zhang ZL, Luo XG, Zhou Q. DRCW-FRkNN-OVO: distance-based related competence weighting based on fixed radius k nearest neighbour for one-vs-one scheme. Int J Mach Learn Cybernet. 2022;13(5):1441–59.

[15] Sen A, Islam MM, Murase K, Yao X. Binarization with Boosting and Oversampling for Multiclass Classification. IEEE Trans Cybern. 2016;46(5):1078–91. 25955858 10.1109/TCYB.2015.2423295

[16] Krawczyk B. Learning from imbalanced data: open challenges and future directions. Progress Artif Intell. 2016;5(4):221–32.

[17] Palli AS, Jaafar J, Hashmani MA, Gomes HM, Gilal AR. A Hybrid Sampling Approach for Imbalanced Binary and Multi-Class Data Using Clustering Analysis. IEEE Access. 2022;10:118639–53.

[18] Pirizadeh M, Farahani H, Kheradpisheh SR. Imbalance factor: a simple new scale for measuring inter-class imbalance extent in classification problems. Knowl Inf Syst. 2023;65(10):4157–83.

[19] Yao L, Lin TB. Evolutionary Mahalanobis Distance-Based Oversampling for Multi-Class Imbalanced Data Classification. Sensors. 2021;21(19):6616. 34640936 10.3390/s21196616 PMC8512012

[20] Ortigosa-Hernández J, Inza I, Lozano JA. Measuring the class-imbalance extent of multi-class problems. Pattern Recognit Lett. 2017;98:32–8.

[21] Zhu R, Wang Z, Ma Z, Wang G, Xue JH. LRID: A new metric of multi-class imbalance degree based on likelihood-ratio test. Pattern Recognit Lett. 2018;116:36–42.

[22] Zhu R, Guo Y, Xue JH. Adjusting the imbalance ratio by the dimensionality of imbalanced data. Pattern Recognit Lett. 2020;133:217–23.

[23] Zhao H, Wu J. Clustering-Based Oversampling Algorithm for Multi-class Imbalance Learning. J Classif. 2025;42(1):205–20. Available from: https://link.springer.com/. .

[24] Stefanowski J. In. Classification of Multi-class Imbalanced Data: Data Difficulty Factors and Selected Methods for Improving Classifiers. 2021. pp. 57–72.

[25] Lango M, Napierala K, Stefanowski J. Evaluating Difficulty of Multi-class Imbalanced Data. In: M. Kryszkiewicz, A. Appice, D. Ślęzak, H. Rybinski, A. Skowron, & Z. W. Raś (Eds.), Foundations of Intelligent Systems. Lecture Notes in Computer Science. 2017;10352:312–22.

[26] Lango M, Stefanowski J. SOUP-Bagging: a new approach for multi-class imbalanced data classification. 2019. Available from: [Cited 14 Oct 2024]. https://www.cs.put.poznan.pl/mlango/publications/SOUPBagging.pdf

[27] Hartono H, Ongko E. Hybrid Approach with Distance Feature for Multi-Class Imbalanced Datasets. JOIV: Int J Inf Visualization. 2023;7(1):131.

[28] Agrawal A, Viktor HL, Paquet E, SCUT. Multi-class imbalanced data classification using SMOTE and cluster-based undersampling. In: 7th International Joint Conference on Knowledge Discovery, Knowledge Engineering and Knowledge Management (IC3K). Lisbon, Portugal: IEEE; 2015. pp. 226–34.

[29] Sanjaya S, Abdillah R, Afrianty I. The impact of Under-Sampling Techniques on Classification Accuracy in multi-class Imbalance Data. In: 2022 3rd International Conference on Electrical Engineering and Informatics (ICon EEI). IEEE; 2022. pp. 92–7.

[30] Arafat MY, Hoque S, Xu S, Farid DM. An Under-Sampling Method with Support Vectors in Multi-class Imbalanced Data Classification. In: 2019 13th International Conference on Software, Knowledge, Information Management and Applications (SKIMA). IEEE; 2019. pp. 1–6.

[31] Krawczyk B, Bellinger C, Corizzo R, Japkowicz N. Undersampling with Support Vectors for Multi-Class Imbalanced Data Classification. In: 2021 International Joint Conference on Neural Networks (IJCNN). IEEE; 2021. pp. 1–7.

[32] Mathew RM, Gunasundari R. A Cluster-based Undersampling Technique for Multiclass Skewed Datasets. Eng Technol Appl Sci Res. 2023;13(3):10785–90.

[33] Abdi L, Hashemi S. To Combat Multi-Class Imbalanced Problems by Means of Over-Sampling Techniques. IEEE Trans Knowl Data Eng. 2016;28(1):238–51.

[34] Kumari A, Thakar U. Hellinger distance based oversampling method to solve multi-class imbalance problem. In: 2017 7th International Conference on Communication Systems and Network Technologies (CSNT). IEEE; 2017. pp. 137–41.

[35] Zhu T, Lin Y, Liu Y. Synthetic minority oversampling technique for multiclass imbalance problems. Pattern Recognit. 2017;72:327–40.

[36] Yang X, Kuang Q, Zhang W, Zhang G. AMDO: An Over-Sampling Technique for Multi-Class Imbalanced Problems. IEEE Trans Knowl Data Eng. 2018;30(9):1672–85.

[37] Dentamaro V, Impedovo D, Pirlo G. LICIC: Less Important Components for Imbalanced Multiclass Classification. Information. 2018;9(12):317.

[38] Krawczyk B, Koziarski M, Wozniak M. Radial-Based Oversampling for Multiclass Imbalanced Data Classification. IEEE Trans Neural Netw Learn Syst. 2020;31(8):2818–31. 31247563 10.1109/TNNLS.2019.2913673

[39] Sridhar S, Kalaivani AA, Two-Tier. Iterative Ensemble Method To Tackle Imbalance In Multiclass Classification. In: 2020 International Conference on Decision Aid Sciences and Application (DASA). IEEE; 2020. pp. 1248–54.

[40] Sridhar S, Kalaivani A. Performance Analysis of Two-Stage Iterative Ensemble Method over Random Oversampling Methods on Multiclass Imbalanced Datasets. Int J Image Graph. 2022;22:02.

[41] Liu M, Dong M, Jing C. A modified real-value negative selection detector-based oversampling approach for multiclass imbalance problems. Inf Sci (N Y). 2021;556:160–76.

[42] Li A, Ma T, Ye S, Liu X, SMOTE-IF:. A Novel Resampling Method Based on SMOTE Using Isolation Forest Variants for Multi-Class Imbalanced Data. In: 2023 IEEE International Conferences on Internet of Things (iThings) and IEEE Green Computing & Communications (GreenCom) and IEEE Cyber, Physical; Social Computing (CPSCom) and IEEE Smart Data (SmartData) and IEEE Congress on Cybermatics (Cybermatics). IEEE; 2023. pp. 570–7.

[43] Tsai CF, Chen KC, Lin WC. Feature selection and its combination with data over-sampling for multi-class imbalanced datasets. Appl Soft Comput. 2024;153:111267.

[44] Prachuabsupakij W, Soonthornphisaj N. Clustering and Combined Sampling Approaches for Multi-class Imbalanced Data Classification. Advances in Information Technology and Industry Applications Lecture Notes in Electrical Engineering. Berlin, Heidelberg: Springer; 2012. pp. 717–24.

[45] Janicka M, Lango M, Stefanowski J. Using Information on Class Interrelations to Improve Classification of Multiclass Imbalanced Data: A New Resampling Algorithm. Int J Appl Math Comput Sci. 2019;29(4):769–81.

[46] Zheng Z, Yan Y, Zhang Y, Zhang Y. Combating Mutuality with Difficulty Factors in Multi-class Imbalanced Data: A Similarity-based Hybrid Sampling. In: 2022 IEEE 9th International Conference on Data Science and Advanced Analytics (DSAA). IEEE; 2022. pp. 1–10.

[47] Munguía Mondragón JC, Rendón Lara E, Alejo Eleuterio R, Granda Gutirrez EE. Del Razo López F. Density-Based Clustering to Deal with Highly Imbalanced Data in Multi-Class Problems. Mathematics. 2023;11(18):4008.

[48] Naglik I, Lango M. GMMSampling: a new model-based, data difficulty-driven resampling method for multi-class imbalanced data. Mach Learn. 2023;113(8):5183-202.

[49] Hoens TR, Qian Q, Chawla NV, Zhou ZH. In.Building Decision Trees for the Multi-class Imbalance Problem. In: Tan, PN., Chawla, S., Ho, C.K., Bailey, J. (eds) Advances in Knowledge Discovery and Data Mining. PAKDD 2012. Lecture Notes in Computer Science. 2012;7301:122-34.

[50] Dong M, Liu M, Jing C. One-against-all-based Hellinger distance decision tree for multiclass imbalanced learning. Front Inform Technol Electron Eng. 2022;23(2):278–90.

[51] Sridhar S, Anusuya S. A dual algorithmic approach to deal with multiclass imbalanced classification problems. Big Data Res. 2024;38:100484.

[52] Cao P, Li B, Zhao D, Zaiane O. A novel cost sensitive neural network ensemble for multiclass imbalance data learning. In: The 2013 International Joint Conference on Neural Networks (IJCNN). IEEE; 2013. pp. 1–8.

[53] Zhou Z, Liu X, ON MULTI-CLASS COST‐SENSITIVE. Learn Comput Intell. 2010;26(3):232–57.

[54] Phoungphol P, Zhang Y, Zhao Y, Srichandan B. Multiclass SVM with ramp loss for imbalanced data classification. In: 2012 IEEE International Conference on Granular Computing. IEEE; 2012. pp. 376–81.

[55] Nababan AA, Nababan EB, Zarlis M, Wage SA, Cost-Sensitive K-. (K-NN) Classification in Handling Multi-class Imbalance Problem. Internetworking Indonesia Journal. 2021;13(2):17–21. Available from: https://internetworkingindonesia.org/index.php/iij/article/view/60. [Cited 03 May 2025].

[56] Febriantono MA, Hadi Pramono S, Rahmadwati R, Naghdy G. Classification of multiclass imbalanced data using cost-sensitive decision tree C5.0. IAES Int J Artif Intell (IJ-AI). 2020;9(1):65.

[57] Azari A, Janeja VP, Levin SMILES. Multiclass Imbalanced Learning in Ensembles through Selective Sampling. In: Proceedings of the Symposium on Applied Computing. New York, NY, USA: ACM; 2017. pp. 811–6.

[58] Lango M, Stefanowski J. Multi-class and feature selection extensions of Roughly Balanced Bagging for imbalanced data. J Intell Inf Syst. 2018;50(1):97–127.

[59] Abdi L, Hashemi S. To combat multi-class imbalanced problems by means of over-sampling and boosting techniques. Soft comput. 2015;19(12):3369–85.

[60] Rodríguez JJ, Díez-Pastor JF, Arnaiz-González Á, Kuncheva LI. Random Balance ensembles for multiclass imbalance learning. Knowl Based Syst. 2020;193:105434.

[61] So B, Valdez EA. SAMME.C2 algorithm for imbalanced multi-class classification. Soft comput. 2024;28(17–18):9387–404. Available from: https://link.springer.com/. .

[62] García S, Zhang ZL, Altalhi A, Alshomrani S, Herrera F. Dynamic ensemble selection for multi-class imbalanced datasets. Inf Sci (N Y). 2018;445–446:22–37.

[63] Hartono H, Risyani Y, Ongko E, Abdullah D. HAR-MI method for multi-class imbalanced datasets. TELKOMNIKA (Telecommunication Comput Electron Control). 2020;18(2):822.

[64] Hartono LS, Rahmadsyah A, Maya Faza Lubis R, Gunawan M. HAR-MI with COSTE in Handling Multi-Class Imbalance. In: 2020 8th International Conference on Cyber and IT Service Management (CITSM). IEEE; 2020. pp. 1–4.

[65] Hartono H, Ongko E. Combining Hybrid Approach Redefinition-Multiclass Imbalance (HAR-MI) and Hybrid Sampling in Handling Multi-Class Imbalance and Overlapping. JOIV: Int J Inf Visualization. 2021;5(1):22–6.

[66] Ongko E, Hartono H. Hybrid approach redefinition-multi class with resampling and feature selection for multi-class imbalance with overlapping and noise. Bull Electr Eng Inf. 2021;10(3):1718–28.

[67] Sridhar S. Anusuya. A Hybrid Approach to Classify the Multiclass Imbalanced Datasets. In: 2023 5th International Conference on Inventive Research in Computing Applications (ICIRCA). IEEE; 2023. pp. 1084–9.

[68] Sun Y, Kamel M, Wang Y. Boosting for Learning Multiple Classes with Imbalanced Class Distribution. In: Sixth International Conference on Data Mining (ICDM’06). IEEE; 2006. pp. 592–602.

[69] Mosley L. A balanced approach to the multi-class imbalance problem. [Ames]: Iowa State University, Digital Repository; 2013.

[70] Hand DJ, Till RJ. A Simple Generalisation of the Area Under the ROC Curve for Multiple Class Classification Problems. Mach Learn. 2001;45(2):171–86.

[71] Mortaz E. Imbalance accuracy metric for model selection in multi-class imbalance classification problems. Knowl Based Syst. 2020;210:106490.

[72] Aguilar-Ruiz JS, Michalak M. Multiclass Classification Performance Curve. IEEE Access. 2022;10:68915–21.

[73] Aguilar-Ruiz JS, Michalak M. Classification performance assessment for imbalanced multiclass data. Sci Rep. 2024;14(1):10759. 38730045 10.1038/s41598-024-61365-z PMC11087593

[74] Du Lmin, Xu Y, Zhu H. Feature Selection for Multi-Class Imbalanced Data Sets Based on Genetic Algorithm. Annals Data Sci. 2015;2(3):293–300.

[75] Li S, Song L, Wu X, Hu Z, Cheung Y, ming YX. Multi-Class Imbalance Classification Based on Data Distribution and Adaptive Weights. IEEE Trans Knowl Data Eng. 2024;36(10):5265–79.

